# High-Intensity Jump Training Is Tolerated during 60 Days of Bed Rest and Is Very Effective in Preserving Leg Power and Lean Body Mass: An Overview of the Cologne RSL Study

**DOI:** 10.1371/journal.pone.0169793

**Published:** 2017-01-12

**Authors:** Andreas Kramer, Jakob Kümmel, Edwin Mulder, Albert Gollhofer, Petra Frings-Meuthen, Markus Gruber

**Affiliations:** 1 Sensorimotor Performance Lab, University of Konstanz, Konstanz, Germany; 2 Institute of Aerospace Medicine, German Aerospace Center (DLR), Cologne, Germany; 3 Department of Sports and Sports Science, University of Freiburg, Freiburg, Germany; University e-Campus, ITALY

## Abstract

**Purpose:**

Space agencies are looking for effective and efficient countermeasures for the degrading effects of weightlessness on the human body. The aim of this study was to assess the effects of a novel jump exercise countermeasure during bed rest on vitals, body mass, body composition, and jump performance.

**Methods:**

23 male participants (29±6 years, 181±6 cm, 77±7 kg) were confined to a bed rest facility for 90 days: a 15-day ambulatory measurement phase, a 60-day six-degree head-down-tilt bed rest phase (HDT), and a 15-day ambulatory recovery phase. Participants were randomly allocated to the jump training group (JUMP, n = 12) or the control group (CTRL, n = 11). A typical training session consisted of 4x10 countermovement jumps and 2x10 hops in a sledge jump system. The training group had to complete 5–6 sessions per week.

**Results:**

Peak force for the reactive hops (3.6±0.4 kN) as well as jump height (35±4 cm) and peak power (3.1±0.2 kW) for the countermovement jumps could be maintained over the 60 days of HDT. Lean body mass decreased in CTRL but not in JUMP (-1.6±1.9 kg and 0±1.0 kg, respectively, interaction effect p = 0.03). Resting heart rate during recovery was significantly increased for CTRL but not for JUMP (interaction effect p<0.001).

**Conclusion:**

Participants tolerated the near-daily high-intensity jump training and maintained high peak forces and high power output during 60 days of bed rest. The countermeasure was effective in preserving lean body mass and partly preventing cardiac deconditioning with only several minutes of training per day.

## Introduction

To maintain crew health during long-term space missions, adequate countermeasures are necessary. Ideally, an exercise-based countermeasure should preserve the integrity and function of bones, neuromuscular and cardiovascular system. In addition, the countermeasure should also be efficient, requiring a minimum of crew time. Currently, astronauts on board the International Space Station are required to exercise for two to two and a half hours per day [1] using three different countermeasure systems (treadmill, cycle ergometer and the advanced resistive exercise device ARED), but even this time-consuming training is not successful in fully maintaining muscle and bone mass and function [1, 2]. As such, space agencies are highly interested in more effective and efficient integrated countermeasures. One type of exercise that has the potential to fulfill these criteria is jumping: jumping is a high-intensity, low-volume type of training that does not require much time, yet, jumping induces high strain and strain rates [3], which have been suggested to be key determinants for bone strength [4, 5]. In addition, jump training has repeatedly been shown to increase leg muscle strength [6] and is associated with increased lean mass [7].

The efficacy of potential countermeasures for spaceflight applications is usually tested in bed rest studies, which serve as a ground-based model for many of the deconditioning effects of microgravity [8, 9]. In the bed rest study described in this paper—organized by the European Space Agency (ESA) and hosted by the German Aerospace Center (DLR)–twelve teams of researchers were involved, using the study as an opportunity to study among others cardiovascular, cognitive, muscular and genetic adaptations to bed rest. The main goal however was to assess the efficacy of a near-daily jump training program (5–6 times per week) during 60 days of bed rest as a countermeasure for muscle and bone loss. The training device used in the present study allows jump training in a supine position and was validated in various previous studies [10–12].

In this manuscript, we will provide a detailed description of the overall experimental scenario of the bed rest study, including the study design, the training program and the participants’ performance during the training. Furthermore, part of the ESA standardized bed rest core data are also presented, including results on body mass, body composition, and vital signs such as resting heart rate, blood pressure and body temperature. In light of the reported efficacy of jump training and the positive effects of physical exercise on cardiovascular health during bed rest [13, 14], we hypothesized that the novel jump training protocol would not only be tolerated during bed rest, as measured by compliance and training performance, but jump training would also maintain lean body mass and prevent deteriorations of vital signs during bed rest.

## Methods

### Study design

This randomized controlled training study was conducted in the: envihab facility of the Institute of Aerospace Medicine at the German Aerospace Center (DLR) in Cologne, Germany. The study was split into two campaigns with initially 12 participants each. One subject dropped out during the second campaign for medical reasons not related to the study, reducing the second campaign to 11 participants. The first campaign started in August 2015, the second campaign in January 2016. Each campaign consisted of 15 days of baseline data collection (BDC-15 through BDC-1), 60 days of HDT bed rest (HDT1 through HDT60) and 15 days of recovery (R+0 through R+14).

### Subject recruitment

Basic inclusion criteria were as follows: male, age between 20 and 45 years, body mass index between 20–26 kg/m2, non-smoking, no medication, no competitive athlete, and no history of bone fractures. After passing an information session and a first psychological test, potential volunteers were invited to a medical screening with clearly defined medical exclusion criteria such as: chronic hypertension, diabetes, obesity, arthritis, hyperlipidemia, hepatic disease (A, C), or a disorder of calcium or bone metabolism. Heritable blood clotting disorders (AT III, S-Akt, Lupus-PTT, ferritin, Factor V Leiden, Factor IV, and Factor II) were also screened for and subject exclusion followed if they had positive test results. Volunteers that were medically eligible for the study subsequently underwent psychological screening, involving questionnaires and interviews. The recruitment process was concluded by a dual energy X-ray absorptiometry (DEXA) screening of the bone mineral density of the femur and the lumbar vertebra column. Of 494 original volunteers, 121 took part in an information session; 60 were medically screened; 45 participated in the psychological interview; and 31 underwent the screening DEXA scan.

### Subjects

Of the 27 volunteers who passed the screening process successfully, 24 were enrolled in the study. One subject discontinued the study on BDC-4 of the second campaign for medical reasons unrelated to the study. In the morning of HDT1, subjects were randomly allocated to either the jump training group (JUMP, n = 12, age 30±7 years, height 181±7 cm and body mass 77±7 kg) or the control group (CTRL, n = 11, age 28±6 years, height 181±5 cm and weight 76±8 kg). Two of the 23 subjects that completed the study (one CTRL, one JUMP) were re-ambulated after respectively 49 and 50 instead of 60 days of HDT due to medical reasons that did not affect their ability to complete the study further nominally (i.e., they were reambulated sooner, but completed the recovery phase with all the scheduled measurements). Consequently, they were only excluded from analyses if they had fewer data points than the other subjects (e.g., daily measurements such as body mass or vital signs). Before taking part in the study, all participants gave written informed consent to the experimental procedures, which were approved by the ethics committee of the Northern Rhine Medical Association (Ärztekammer Nordrhein) in Duesseldorf, Germany, as well as the Federal Office for Radiation Protection (Bundesamt für Strahlenschutz). Subjects received a substantial financial reward for participating in the study (15’000€ for the whole study, including follow-up measurements).

### Bed rest routine

Each subject was accommodated in a single-person room, equipped with television, telephone, and laptop with internet access. Room temperature within the ward and rooms was kept at 20–23°C, humidity at 30–50%. During the bed rest period (starting at 9 A.M. of HDT1), the subjects maintained the 6° HDT for 24 h/day. All personal hygiene activities (bowel movement, showering etc.) were performed in the HDT position. Round-the-clock staff monitoring ensured compliance with the protocol. The subjects were not allowed to elevate their heads more than 30° from horizontal. Horizontal displacements were allowed, but static and dynamic muscle contractions were strictly prohibited. During the adaptation and recovery phases (BDC and R), physical activity was restricted to free movement within the ward.

To ensure safety and well-being of subjects, a 24h/day medical and paramedical care was provided. As part of this standardized procedure, general health indicators such as blood pressure and heart rate were assessed daily (Intellivue MMS X2, Philips, Best, The Netherlands) in the fasting state, immediately following the scheduled wake-up at 6:30 AM (lights off at 11 PM). Body mass (BM) was assessed daily following the first urine void of the day (DVM 5703, Sartorius, Goettingen, Germany). 24h urine volume pools were also assessed daily. Safety parameters (from blood and urine samples) were periodically assessed by an independent medical doctor, who additionally monitored the subjects’ health status during daily ward rounds. Psychological support by an independent psychologist was provided during weekly individual support talks. Finally, the questionnaires that aimed to assess the acceptability of the countermeasure (modified Nordic Musculoskeletal Questionnaire, and Physical Activity Enjoyment Scale) were obtained at weekly intervals, and subjects were required to keep a daily log of critical incidents.

### Blood volume

Blood volume was determined before bed rest and on the final day of bed rest using the optimized Carbon Monoxide Rebreathing Technique (CORT). In brief, after an initial resting period in the supine position for 20 min during BDC and 6° HDT during HDT60, a baseline 3-ml EDTA blood sample was obtained from an antecubital vein (S-Monovette^®^, Sarstaedt AG & Co., Nümbrecht, Germany) that was immediately placed on ice for subsequent analyzes. The subjects were then connected to a Krogh-spirometer (Student Spirometer, ZAK, Germany) and started the rebreathing procedure: after a complete exhalation, subjects completely inhaled a ~3 l bag containing pure oxygen. A bolus of ~70 ml CO was simultaneously applied (1.0 ml kg body mass for trained males, 0.8 ml kg body mass for untrained males (less than 2h of physical activity per week)). After an initial 10 s breath-hold, subjects remained breathing through the mouthpiece until two minutes of CO-rebreathing were completed. Five minutes after the termination of the rebreathing, a second 3-ml EDTA blood sample was obtained. Blood gas and blood count analyses were then immediately performed via routine clinical work using the ABL 520, Radiometer, Denmark, and the ABX Pentra 60 hematology analyzer (Horiba ABX SAS, Montpellier Cedex, France).

### DEXA measurements

Body composition was assessed with a Prodigy Full Pro (GE Healthcare GmbH, Solingen, Germany) using the whole body scan feature, and the manufacturer’s enCORE software (version 16.10.151) was used to generate automated reports on total bone mineral content, total fat and total lean tissue mass.

### Diet

During the entire study, the subjects received a strictly controlled and individually tailored diet. The individual energy intake (total energy expenditure, TEE) was calculated by multiplying resting metabolic rate by a factor of 1.6 during the ambulatory phases (BDC and R) and a factor of 1.3 during HDT for CTRL and 1.33 for JUMP (to compensate for the higher energy requirements during the training, based on spirometry data collected during a pilot study). Resting metabolic rate was measured on BDC-14 by indirect calorimetry using the Weir Equation, assuming 8.64 g/d for Urinary Nitrogen (metalyzer 3B, CORTEX Biophysik GmbH). Daily body weight, 24h urine pools, and body composition, as biweekly measured by DEXA were used to monitor the validity of the diet. In case of systemic changes in total body fat mass that exceeded 1kg, the dietary total energy intake was slightly adjusted, whilst leaving the relative contributions of the macronutrients constant.

Total energy intake was determined individually for each participant, discriminating between protein, fat, carbohydrates and fibers as follows: the energy intake of proteins was set at 1.2g/kg/d; the energy intake of fat was set at 35% of the total intake; the energy intake of fibers was set at minimally 30 g/d; finally, the energy intake of carbohydrates was determined by the total energy intake minus the energy provided by fat, fiber and protein intake. Thus, the relative intake levels of protein, fat, carbohydrates and fibers, were approximately 14%, 35%, 49%, and 2% (for details see Table 1).

**Table 1:** Average daily energy, water, minerals and vitamin intake during the baseline data collection phase (BDC), head-down tilt bed rest (HDT, separately for the training group (JUMP) and the control group (CTRL)) and recovery.

	BDC	HDT bed rest	HDT bed rest	Recovery
		CTRL	JUMP	
Energy (kcal/d)	2688 ± 267	2080 ± 222	2066 ± 129	2692 ± 239
Protein (g/d)	92 ± 8	92 ± 9	93 ± 8	92 ± 8
Protein (g/kgBW/d)	1.21 ± 0.01	1.21 ± 0.01	1.20 ± 0.02	1.21 ± 0.01
Protein (%TEE/d)	14 ± 1	18 ± 2	19 ± 1	14 ± 1
Fat (g/d)	102 ± 10	79 ± 8	78 ± 5	102 ± 9
Fat (%TEE/day)	35 ± 0	35 ± 0	35 ± 0	35 ± 0
Carbohydrates (g/d)	332 ± 39	236 ± 33	231 ± 17	334 ± 34
Carbohydrates (%TEE/day)	51 ± 1	46 ± 2	46 ± 1	51 ± 1
Fiber (g/d)	31 ± 1	33 ± 2	32 ± 1	32 ± 1
Fluid (g/d)	3820 ± 338	3830 ± 390	3851 ± 324	4239 ± 441
Fluid (ml/kgBW/d)	50.0 ± 0.3	50.5 ± 0.7	50.0 ± 0.1	56.0 ± 6.0
Calcium (mg/d)	1096 ± 44	1117 ± 43	1123 ± 46	1106 ± 38
Chloride (mg/d)	5914 ± 496	5856 ± 576	5932 ± 502	5856 ± 502
Chloride (mmol/kgBW/d)	2.18 ± 0.03	2.17 ± 0.01	2.17 ± 0.02	2.17 ± 0.03
Potassium (mg/d)	4215 ± 280	4149 ± 249	4068 ± 248	4193 ± 318
Sodium (mg/d)	3866 ± 336	3752 ± 378	3811 ± 320	3846 ± 340
Sodium (mmol/kgBW/d)	2.20 ± 0.03	2.15 ± 0.01	2.15 ± 0.01	2.20 ± 0.03
Magnesium (mg/d)	422 ± 18	443 ± 18	444 ± 19	418 ± 18
Phosphorus (mg/d)	1634 ± 100	1598 ± 118	1614 ± 100	1600 ± 115
Iron (mg/day)	13.5 ± 0.7	15.3 ± 0.8	15.2 ± 0.7	13.7 ± 1.2
Fluoride (μg/d)	2150 ± 203	2400 ± 183	2470 ± 234	2144 ± 267
Jodide (μg/d)	249 ± 23	271 ± 25	275 ± 23	225 ± 26
Zinc (mg/d)	12.7 ± 0.7	12.6 ± 0.5	12.6 ± 0.5	12.9 ± 0.7
Copper (μg/d)	2046 ± 137	1840 ± 140	1840 ± 115	2092 ± 204
Biotin (μg/d)	59.2 ± 3.1	70.1 ± 3.8	70.4 ± 3.7	59.5 ± 3.8
Folic acid (μg/d)	736 ± 54	853 ± 48	864 ± 63	735 ± 68
Niacinequivalent (μg/d)	30586 ± 2341	29263 ± 2651	29591 ± 2543	30087 ± 2564
Pantothenicacid (mg/d)	6.5 ± 0.4	6.3 ± 0.4	6.3 ± 0.4	6.6 ± 0.6
Retinolequivalent (μg/d)	1894 ± 140	3005 ± 194	2833 ± 200	1894 ± 192
Vit A Retinol (mg/d)	1 ± 0	1 ± 0	1 ± 0	1 ± 0
Vit B1 (mg/d)	1.5 ± 0.1	1.7 ± 0.1	1.7 ± 0.1	1.5 ± 0.2
Vit B12 (μg/d)	5.5 ± 0.6	5.5 ± 0.5	5.6 ± 0.6	5.5 ± 0.5
Vit B2 (mg/d)	1.8 ± 0.1	1.9 ± 0.1	1.9 ± 0.1	1.8 ± 0.2
Vit B6 (mg/d)	2.2 ± 0.2	2.3 ± 0.2	2.3 ± 0.2	2.2 ± 0.2
Vit C (mg/d)	258 ± 28	242 ± 18	240 ± 17	252 ± 27
Vit D (IU/d)	1133 ± 15	1125 ± 27	1131 ± 34	1138 ± 55
Vit E (mg/d)	16.2 ± 0.9	17.1 ± 1.8	16.9 ± 1.2	16.6 ± 2.5
Vit K (μg/d)	221 ± 12	381 ± 34	375 ± 40	224 ± 12
Potential renal acid load	-8 ± 5	-9 ± 7	-6 ± 2	-9 ± 6

The daily diet was also constant for water intake (50 mL/kgBM). Additional fluid and energy intake was administered in the form of water and diluted apple juice following physically demanding experiments to compensate for sweat and energy loss. During the first few days of recovery (R+0 through R+5), water intake had no upper limit to compensate the reambulation-induced fluid-shift. Intake of caffeine, as well as alcohol consumption, was not allowed. In order to account for the lack of sunlight exposure, the subjects were supplemented with 1000 IU/d of vitamin D, during the study (see Table 1). Vitamins and elements were controlled and standardized as well and achieved as a minimum the recommended Dietary Reference Intakes (http://ods.od.nih.gov). One subject (H) was found to have an iron deficiency during the pre-study screening and was supplemented for 8 weeks prior to study start (2 x day 50mg Ferro sanol duodenal). All meals for this study were prepared in a metabolic kitchen where all foods were weighed to ± 0.5 g using laboratory scales. The nutrient content of each prepared meal was calculated, using the PRODI software (Kluthe Prodi 6.3 ^®^ expert).

### Physiotherapy

Physiotherapy was implemented weekly during the first campaign starting on HDT6. After three weeks of bed rest, physiotherapy was increased to twice per week, primarily for psychological reasons, as the subjects enjoyed the variety, especially the control subjects. In addition, with two-weekly visits, the physiotherapists could respond more quickly to specific physiotherapy needs. To alleviate back pain during the early days of the bed rest, in campaign 2, additional physiotherapy sessions were scheduled daily between HDT2 and HDT5 and subsequently twice per week. The main objective of the physiotherapy sessions was the passive movement of the joints of the lower extremity. In addition, subject underwent a light massage with little pressure of the back to release muscle tension. At the cervical spine, traction was implemented to relieve the spinal discs. In addition, individual therapies were implemented in case of discomfort (e.g. tension headaches, or sacroiliac joint blockages).

### Reconditioning

During the BDC phase (BDC-11 or BDC-10), subjects were familiarized with the exercises they would encounter following re-ambulation. As for the actual reconditioning, a total of six 30-minute sessions were scheduled between R+2 and R+14 for each subject. Only one session (i.e. only 1%) was not performed due to foot soreness (subject F). Reconditioning consisted of active stretching, fast footwork with an “agility ladder”, and exercises on a Bosu ball (Bosu, Ashland, Ohio, USA). The reconditioning was multifocal and aimed at increasing range of motion, improving muscle strength, speed and coordination as well as core stability and body posture. The exercises started slow and easy, and then progressed in movement speed and difficulty, tailored to each subject’s status.

### Training device

**Fig 1:**
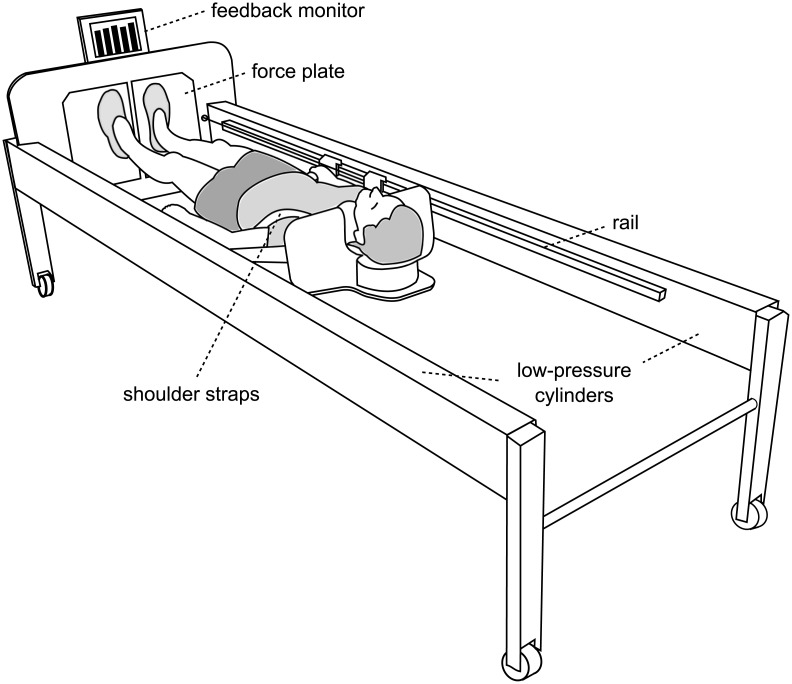
The training device (sledge jump system, SJS). The participant is fixed to the wooden sledge with shoulder straps, and his thighs rest on additional straps. The straps are attached to the rails and can slide in the direction of the rails with minimal friction. The participant stands on two force plates (separated, one for each foot).

**Fig 2:**
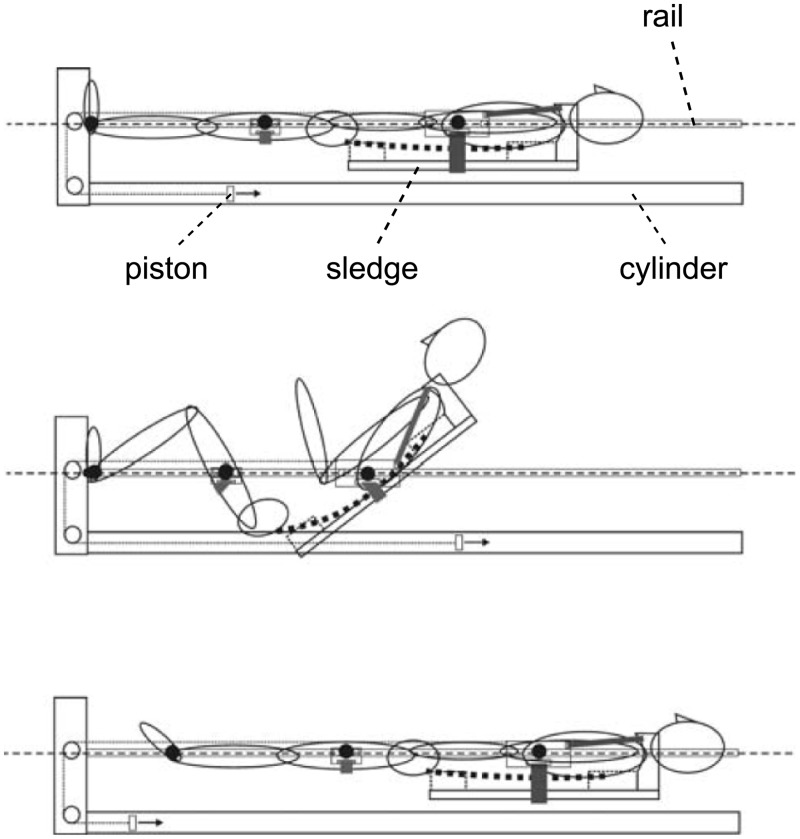
A longitudinal section of the SJS. The two low-pressure cylinders pull the participant onto the force plates via ropes attached to the wooden sledge. The second drawing illustrates the freedom of movement in the ankle, knee and hip joint.

The sledge jump system (SJS, see Fig 1) was developed by Novotec Medical GmbH (Pforzheim, Germany). It consists of a frame on wheels and a lightweight sledge (5 kg) that is attached to a rail on both sides of the frame. The construction allows the sledge only to slide alongside the rails. Note though that the sledge is attached to the rails with straps that allow some movement perpendicular to the movement direction as well as some rotation (see Fig 2). The participant is attached to the sledge via two straps around the shoulders, allowing movement in a natural manner [10]. The force that pulls the sledge towards the force plates is generated by four low-pressure cylinders. One cylinder can generate 450 N at full capacity, i.e. any force between zero and 1800 N can be set by altering the pressure of the cylinders. Ground reaction forces were recorded via two force plates, and the position of the sledge was recorded via an incremental encoder. A feedback monitor at the bottom of the SJS was used to provide live feedback (e.g., jump height or peak force) for every single jump.

### Familiarization

During nine 30-minute sessions (BDC-15 through BDC-7), all subjects were familiarized with the correct jumping technique in the SJS. Each familiarization session consisted of a warm-up (mobilization of the ankle joints, six deep static squats, six heel raises, three submaximal CMJs, one series of submaximal hops) and then six countermovement jumps (CMJ) and four series of ten hops each. The force in the SJS was gradually increased from 50% body weight in the first session up to 100% body weight in the last session. Participants were shown the correct jumping technique and received verbal feedback about their technique after each series of jumps. In addition, visual feedback about the target parameters (peak force for the hops and jump height for the CMJs) was provided for each jump on the SJS feedback monitor.

### Training

**Fig 3:**
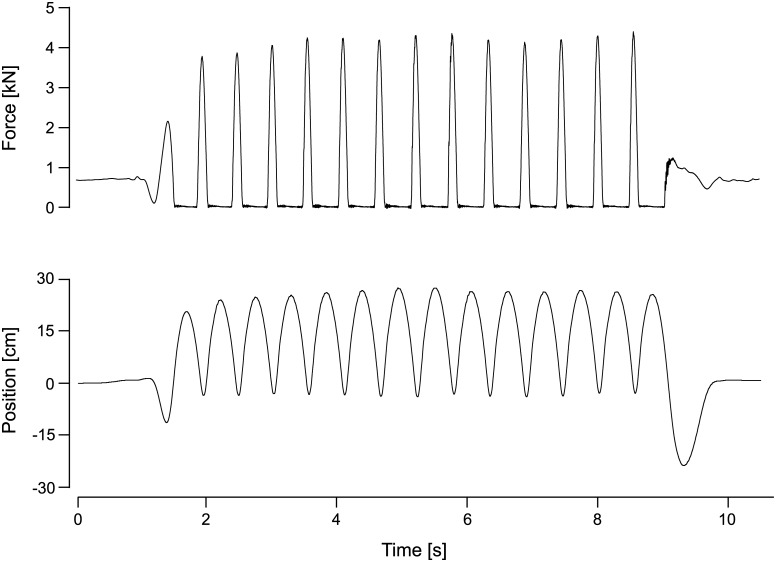
Training data. Exemplary ground reaction forces and position signal from one series of 13 hops during one participant’s training session in the SJS.

The training protocol implemented in the study was finalized based on a five-week preparatory training study at the University of Konstanz (Germany) that included 26 subjects and 27 sessions per subject. The final training protocol for the JUMP group during the 60 days of HDT comprised a total of 48 training sessions (6x type A, 15x type B, 14x type C, 13x type D). The details of the four different training sessions (A to D) are shown in Table 2. Essentially, each session consisted of a varying number of CMJs and repetitive hops, preceded by a warm-up (see familiarization) and three maximal CMJs at 80% BW. The instruction for the hops was as follows: “Jump as stiff as possible, i.e. flex the ankle, knee and hip joint as little as possible while still jumping as high as the high stiffness allows; do not let the heels touch the plate during landing, keep the contact time as short as possible and jump as constant as possible.” For the CMJs, it was “Quickly drop to a squat and then immediately jump as high as possible”. These instructions were repeated and the correct execution demonstrated whenever necessary. All sessions were supervised and documented, and subjects were given verbal encouragement during the exercises. Ground reaction forces as well as sledge position data were recorded for each jump. The force and position data from one exemplary series of hops is shown in Fig 3.

**Table 2:** Details of each of the four different training sessions. Average load refers to the pre-set force in the training device, expressed in percent of bodyweight.

Training type	A	B	C	D
Amount of hops	2 x 12	2 x 12	2 x 15	4 x 15
Amount of CMJs	10 x 3	4 x 10	2 x 15, 2 x 20	1 x 12
Average load	85% BW	90% BW	80% BW	80% BW
Breaks between series	1’	1’30”	30”	1’
Total training duration	17’	14’30”	9’30”	8’30”
Duration excluding breaks	3’	4’	4’	1’30”

### Data processing and statistics

The peak force of the hops was averaged over all the hops performed during one training session. For the CMJs, the jump height was directly determined based on the position signal of the sledge. Peak power was calculated as the product of ground reaction force and the velocity of the sledge (derivative of the position signal). To assess differences between the jump performance at the beginning of HDT and jump performance at the end of HDT, the first and last training session of each of the four types was analyzed and the mean of these first four compared to the mean of the last four using two-tailed t-tests for paired samples (alpha = 0.05, just as for the other analyses).

Changes in body composition were assessed with repeated measures analyses of variance (rmANOVA), using time (BDC-3 and R+7) as repeated measure and group (JUMP, CTRL) as inter-subject factor. Blood parameters (hematocrit, hemoglobin (Hb), total red cell volume (tRCV), mean corpuscular hemoglobin concentration (MCHC), blood volume (BV) and plasma volume (PV)) were assessed with a rmANOVA using time (BDC-7 and HDT60) as repeated measure and group (JUMP, CTRL) as inter-subject factor. The daily general health indicators (morning blood pressure, heart rate, oral temperature, body weight, as well as 24hr urine volume) were also analyzed with rmANOVA, using time (baseline, i.e., mean of BDC-14 through BDC-1, and R+1) as repeated measure and group (JUMP, CTRL) as inter-subject factor. To illustrate the progression during the recovery phase, Bonferroni-corrected two-tailed t-tests for paired samples were used, comparing recovery values to baseline values. The PACES questionnaire contains 18 items on a 5-point Likert scale and was analyzed by summing up all the 18 items after flipping the negative items.

Group data are presented as means ± standard deviations (SD). The analyses were executed either by using SPSS 21.0 (SPSS, Inc., Chicago, IL).

## Results

### Training performance

**Fig 4:**
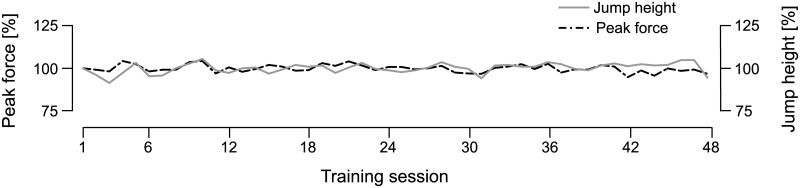
Training performance. Grand mean of the peak forces of the hops and the countermovement jump height throughout the 48 training sessions in the SJS during the 60 days of bed rest. The black broken line represent the averaged peak forces of the hops in the corresponding training session and the grey line represents the averaged countermovement jump height.

All of the key performance parameters of the jump training (peak forces, jump height and peak power) remained constant or increased throughout the 60 days of bed rest: the peak forces during the hops showed no significant changes (3.6±0.4 kN at the beginning of HDT versus 3.6±0.5 kN at the end of HDT, p = 1.0, see Fig 4), and jump height (35±4 cm versus 37±5 cm, p<0.05) as well as peak power for the CMJs increased (3.1±0.2 kW versus 3.4±0.3 kW, p<0.01).

### Adherence and compliance

All subjects completed all of their familiarization and training sessions as scheduled except for one familiarization session due to one subject’s late arrival at DLR. In 42 out of 675 training and familiarization sessions (6%), the subject trained with less than maximal effort due to headache, indisposition or other discomfort (affected body regions: see questionnaire results below). Three training sessions were slightly altered due to discomfort during one jump type (hops instead of CMJs, but amount of jumps unchanged). One participant of the first campaign started in the training group, but was reallocated to the control group after three training sessions due to a possible medial tibia stress syndrome.

### Countermeasure acceptability questionnaires

The grand mean of the Physical Activity Enjoyment Scale score was 72±13 (theoretical maximum: 90 points) and remained fairly constant throughout the training (73±11, 75±10, 73±14, 71±16, 71±16, 70±16, 71±16, 68±16 at weekly intervals). In the weekly modified Nordic Musculoskeletal Questionnaires, seven out of the 23 subjects reported discomfort that on occasion influenced their ability to do the training or familiarization with maximal effort. Affected body regions for the hops were the feet (two subjects), Achilles tendon (one subject with a history of Achilles tendon pain) and lower back (two subjects). For the countermovement jumps the affected regions were the hip/groin (two subjects), the thighs (one subject, muscle soreness) and the neck (one subject).

### DEXA-based body composition, body mass

**Fig 5:**
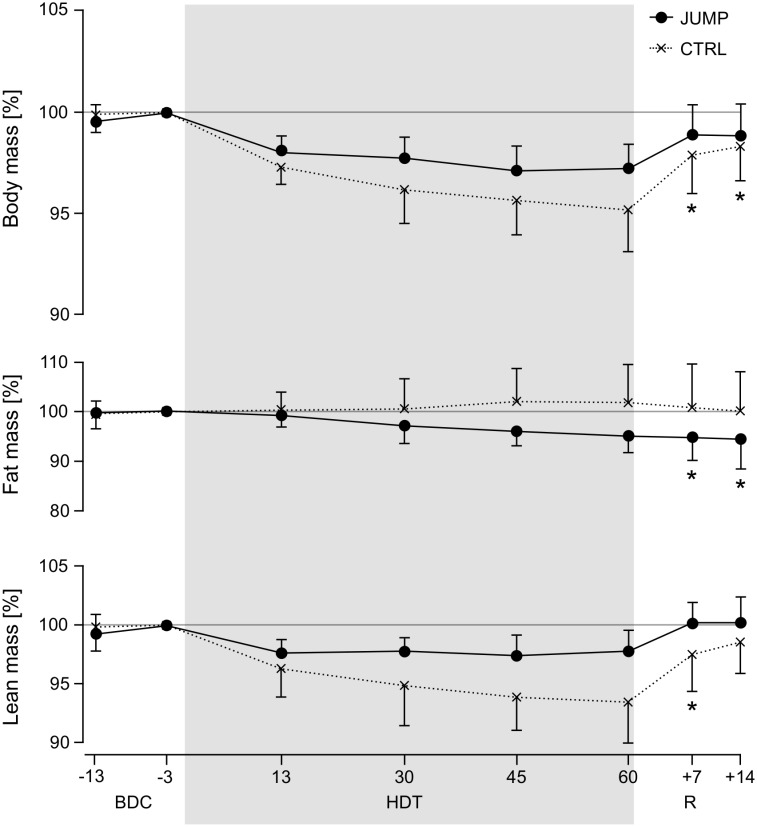
Body composition. Body composition based on DEXA analyses, normalized to BDC-3 values. Solid lines represent mean (and standard deviation) for the training group (JUMP), broken lines for the control group (CTRL). Two measurements were taken during baseline data collection (BDC-13 and BDC-3), four during bed rest (HDT13, HDT30, HDT45, HDT60) and two during recovery (R+7, R+14). Values during the recovery phase that differ significantly from BDC-3 values are marked with an asterisk symbol (separately for CTRL and JUMP).

When comparing the time course of total body mass, fat mass and lean mass between the two groups (see Fig 5 and Table 3), there are some notable differences: participants in CTRL lost lean mass, while the participants in JUMP preserved their lean mass (significant time*group interaction effect of the ANOVA comparing time points BDC-3 and R+7, F_1,21_ = 5.5, p = 0.03). For fat mass, the opposite was found: JUMP showed decreased fat mass, whereas fat mass in CTRL slightly increased (however, no significant time*group interaction effect when comparing time points BDC-3 and R+7, F_1,21_ = 2.2, p = 0.15, only a significant main effect of time, F_1,21_ = 5.0, p = 0.04). There was no interaction (F_1,21_ = 1.7, p = 0.20) or time effect (F_1,21_ = 0.04, p = 0.85) for total bone mineral content. Total body mass (daily scale measurements) decreased less in JUMP compared to CTRL (significant time*group interaction effect when comparing baseline (mean of BDC-14 through BDC-1) and R+0, F_1,19_ = 6.9, p = 0.017, but no significant interaction anymore when comparing baseline to R+1, F_1,19_ = 2.9, p = 0.10). Recovery values were significantly lower compared to baseline for CTRL from R+0 through R+6, whereas no significant differences were found for JUMP apart from R+0 and R+1.

**Table 3:** Results of the DEXA measurements, separately for the training group (JUMP) and the control group (CTRL), once during baseline (BDC-3), once at the end of bed rest (HDT60) and once during recovery (R+7).

	JUMP BDC-3	JUMP HDT60	JUMP R+7	CTRL BDC-3	CTRL HDT60	CTRL R+7
Lean mass [kg]	56.4±5	55.1±4	56.4±5	56.9±7	53.0±5	55.4±6
Fat mass [kg]	19.2±6	18.3±6	18.3±6	16.9±4	17.0±3	16.7±3
BMC [kg]	3.0±0.3	3.0±0.3	3.0±0.3	3.1±0.4	3.1±0.4	3.1±0.4
Total mass [kg]	78.6±7	76.4±6	77.7±7	77.0±8	73.2±7	75.2±7

### Heart rate, blood pressure and 24h urine

**Fig 6:**
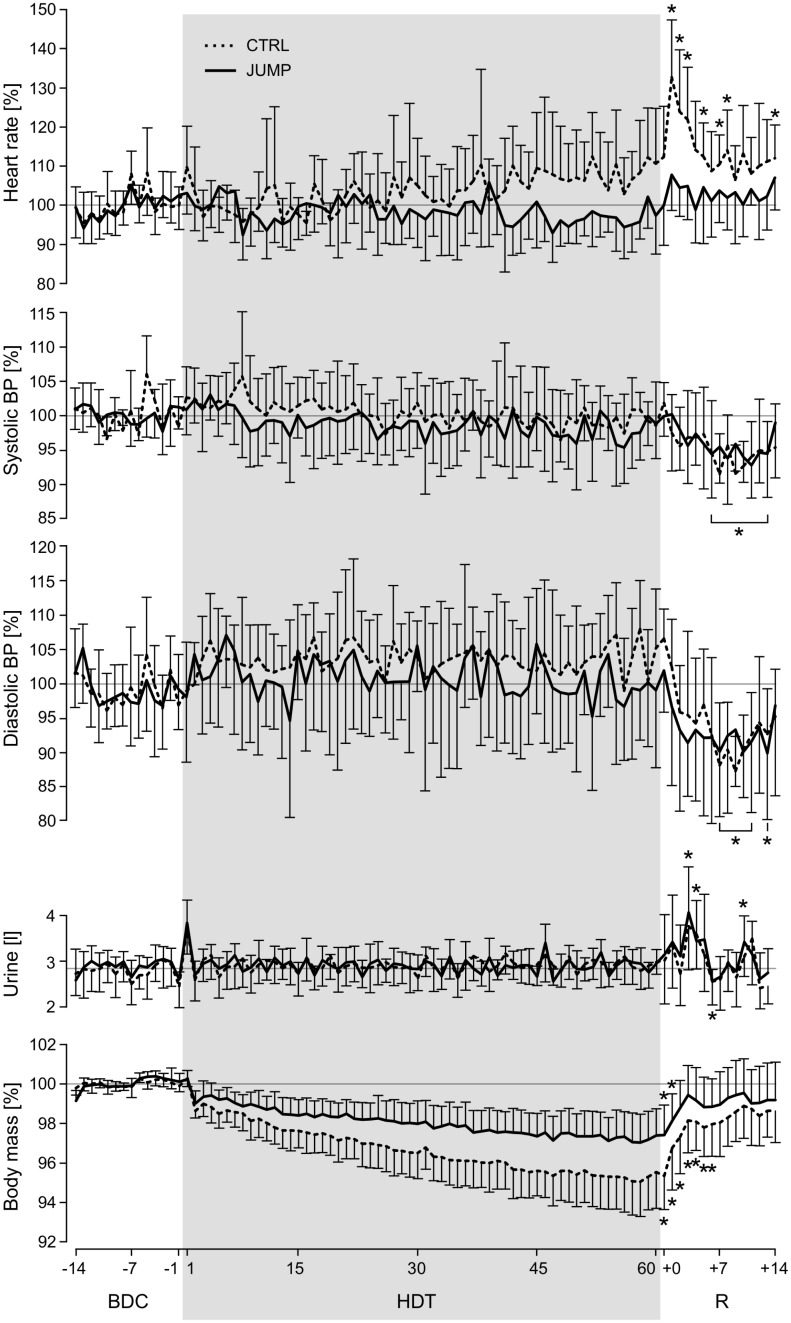
Daily measurements. Vitals, urine volume and body mass measured daily in the morning, normalized to baseline values (mean of BDC-14 through BDC-1). Broken lines represent the mean and standard deviation of the control group, solid lines represent mean and standard deviation of the training group. Values during the recovery phase (R+x) that differ significantly from baseline values are marked with an asterisk symbol (for heart rate and body mass separately for CTRL and JUMP, for blood pressure and urine for all subjects together, as these parameters showed no significant differences between groups). Note that from R+0 until R+5, subjects’ fluid intake had no upper limit.

A significant time*group interaction effect (F_1,19_ = 19.1, p<0.001) was observed for the daily heart rate measurements when comparing baseline to the R+1 values, and for the control group a significantly increased heart rate compared to baseline was observed during recovery (see Fig 6). No significant interaction effects were found for systolic blood pressure (F_1,19_ = 0.4, p = 0.54), diastolic blood pressure (F_1,19_ = 2.2, p = 0.15), oral temperature (F_1,19_ = 2.2, p = 0.54) and 24h urine (F_1,19_ = 0.0, p = 0.99), see Table 4. However, the comparison of the blood pressure for all subjects together during some of the recovery days (starting on R+6) showed a decrease compared to baseline (see Fig 6). 24h urine volume for all subjects was significantly increased on HDT1 as well as during some of the recovery days.

**Table 4:** Results of the daily measurements (heart rate, systolic blood pressure, diastolic blood pressure, urine, oral temperature and body mass), separately for the training group (JUMP) and the control group (CTRL), once during baseline (mean of BDC-14 through BDC-1), once at the end of bed rest (HDT60) and once at the beginning of recovery (R+1).

	JUMP baseline	JUMP HDT60	JUMP R+1	CTRL baseline	CTRL HDT60	CTRL R+1
HR [bpm]	60±8	58±11	64±11	54±8	60±12	72±12
SBP [mmHg]	118±11	117±13	118±13	110±9	109±14	113±10
DBP [mmHg]	65±7	66±11	64±11	61±5	66±8	64±6
Urine [l]	2.9±0.2	3.0±0.3	3.4±1.0	2.8±0.4	3.0±0.3	3.3±0.9
Oral temperature [°]	36.0±0.3	36.1±0.3	36.1±0.2	36.0±0.3	36.0±0.2	36.2±0.4
Body mass [kg]	77.7±6	76.5±6	77.0±6	75.9±8	72.4±7	73.3±7

### Blood volume and blood count

No significant time*group interaction effects were found for any of the analyzed blood parameters when comparing the values measured for BDC-7 to those measured for HDT60 (Table 5). However, there was a significant main effect of time for MCHC (+1±1%, F_1,21_ = 16.1, p = 0.001), tHb (-12±9%, F_1,21_ = 44.2, p<0.001), tRCV (-13±9%, F_1,21_ = 49.8, p<0.001), blood volume (-13±6%, F_1,21_ = 96.3, p<0.001) and plasma volume (-13±6%, F_1,21_ = 102.0, p<0.001), but not for hematocrit (0±5%, F_1,21_ = 0.002, p = 0.96) and Hb (+1±5%, F_1,21_ = 1.0, p = 0.33).

**Table 5:** Blood parameters (hematocrit, hemoglobin (Hb), total red cell volume (tRCV), mean corpuscular hemoglobin concentration (MCHC), blood volume (BV) and plasma volume (PV)), separately for the training group (JUMP) and the control group (CTRL), once during baseline (BDC-7) and once at the end of bed rest (HDT60).

	JUMP BDC-7	JUMP HDT60	CTRL BDC-7	CTRL HDT60	Main effect of time
Hematocrit [%]	42.8±2.4	43.2±2.8	42.9±2.4	42.5 1.7	p = 0.96
Hb [g/dl]	15.1±0.8	15.4±1.0	15.1±0.9	15.2±0.6	p = 0.33
MCHC	35.4±0.4	35.7±0.5	35.3±0.5	35.7±0.5	p = 0.001
tHb [g]	830±132	735±96	877±125	755±95	p<0.001
tRCV [ml]	2348±376	2061±259	2485±347	2116±282	p<0.001
BV [ml]	6013±725	5242±531	6365±806	5478±699	p<0.001
PV [ml]	3665±393	3181±334	3880±508	3362±442	p<0.001

## Discussion

With this short but intensive jump training program, the participants of the training group were able to preserve lean body mass, maintain high peak forces and even increase jump height and power output throughout the 60 days of bed rest.

This is a remarkable result considering the brevity of the training (approx. 3 minutes spent exercising per training session) and the fact that there was no progression of the training volume over the course of the 48 training sessions. Another strength of the countermeasure used in this study is the continuous monitoring of each subject’s training performance, so that every training doubles as a performance test assessing peak power output and peak forces. All in all, the training was well tolerated by the subjects as demonstrated by the perfect adherence rate to the training of 100% (a first for long-term bed rest studies), the high scores on the Physical Activity Enjoyment Scale, and the relatively low number of reported training-attributed discomforts. However, just like for any other type of intensive training, several precautions should be—and were—taken to reduce the risk of injuries: subjects should be familiarized with the correct jump technique in the SJS before the training phase, and in the recruitment process sedentary subjects or subjects with preexisting conditions (especially regarding shoulder, neck, lower back, tibia and Achilles tendon) should be excluded. An additional interesting point concerning lower back pain is that during and some hours after the training, subjects in the training group reported a decrease in the well-known lower back pain experienced by most subjects during the first days of HDT. A possible explanation for this observation is the fact that during the time spent in the training device, the subject’s intervertebral discs are compressed, which counteracts the increased volume of the discs that has been proposed as the cause of lower back pain experienced by bedridden subjects and astronauts alike [15].

The DEXA measurements for body composition show that the training group preserved lean body mass and decreased fat mass, even though their total energy intake compared to the one of the control group was higher to compensate for higher energy expenditure due to exercise. When comparing the first DEXA data point after bed rest to baseline values, it becomes evident that the training group’s loss of 0.9±1.2 kg in total body mass is due to the 0.9±1.0 kg loss in fat mass while lean body mass did not change. The control group’s loss of 1.7±1.6 kg in total body mass on the other hand stems from a 1.6±1.9 kg loss in lean body mass while fat mass was maintained. Note that the training group’s seeming loss in lean body mass at the HDT data points can most likely be attributed to the well-documented increased diuresis on the first day of HDT [16, 17], attributed to the Gauer-Henry-Reflex [18]: on HDT1, 24h urine volume increases by 1.0±0.3 liter, consistent with the drop in body mass by 1.0±0.3 kg from HDT1 to HDT2. The seeming “loss” in lean body mass during HDT also amounts to 1.3±1.1 kg (constant for all HDT data points), which completely disappeared during recovery by reambulation-induced fluid retention. The increased 24h urine volume at the beginning of the recovery phase is a result of the ad libitum increased fluid intake during R+0 through R+5.

The training group and the control group also differed in their response to bed rest and reambulation with respect to resting heart rate: the control group’s heat rate slowly increased from the middle of HDT onwards—which is consistent with the results from previous bed rest studies [14, 19]–and showed another marked increase at the beginning of the recovery phase. The training group, however, showed no increase or even a decrease in heart rate during HDT, and only a slight, statistically not significant increase at the beginning of the recovery phase. Previously, it was reported that an elevated heart rate during or after bed rest was associated with decreased stroke volume, decreased cardiac vagal tone, increased sympathetic catecholamine secretion, and greater cardiac beta-receptor sensitivity and an overall decrease in maximal cardiac output [20]. All of these interconnected parameters can be influenced by exercise [21], making it possible that several of them were positively influenced by the countermeasure, counteracting the cardiac deconditioning effects observed in the control group. Note that this difference between groups cannot be explained by changes in plasma volume, which was similar in both groups and comparable to the volume losses reported in earlier studies [17, 22]. Indeed, plasma volume, blood volume, total red cell volume and total hemoglobin decreased by the same amount across groups (-13%), whereas the relative parameters (hematocrit, hemoglobin and mean corpuscular hemoglobin concentration) did not change much (0–1%). Hence, a decrease in blood volume with little change in blood composition seems to have occurred in both groups.

In contrast to resting heart rate, blood pressure at rest showed no differences between the training group and the control group. It remained fairly constant during HDT—also consistent with previous reports [23, 24]–and showed a decrease on several days between R+6 and R+13. This reduction in blood pressure after reambulation has been observed in some previous studies [17] but not consistently [19, 25] and is thought to be primarily caused by the reduction in plasma volume [26]. However, if the decreased blood pressure were only an effect of the decreased plasma volume, one would expect the blood pressure to have decreased already during R+1 through R+5, which was not the case, so more detailed measurements are necessary to elucidate this matter. In addition, a larger sample size would have been helpful to study secondary phenomena such as the slight decrease in blood pressure, but as it is usually the case with expensive and logistically challenging studies such as bed rest studies, sample size estimations were based on the primary endpoints.

From an applied perspective, the results of the study suggest that a training program for astronauts does not need to incorporate high training volumes in order to effectively preserve lean body mass, leg muscle power and important parameters of cardiac function such as resting heart rate. Indeed, the results of the present study reveal that a low-volume, whole-body exercise program involving maximal effort during every repetition seems to be both effective and efficient.

## Conclusion

The participants in the countermeasure group tolerated the near-daily high-intensity jump training well, were able to maintain high peak forces and increase high power output for the jumps during 60 days of bed rest. The jump countermeasure was also very effective in preserving lean body mass and preventing an increase in resting heart rate during bed rest and recovery with only several minutes of training per day. Blood pressure and blood volume followed the dynamics observed in previous bed rest studies, notably a decrease in blood pressure during the recovery phase and a decrease of blood volume after bed rest.
